# Lactate Metabolism Is Strongly Modulated by Fecal Inoculum, pH, and Retention Time in PolyFermS Continuous Colonic Fermentation Models Mimicking Young Infant Proximal Colon

**DOI:** 10.1128/mSystems.00264-18

**Published:** 2019-05-28

**Authors:** Van Thanh Pham, Christophe Chassard, Etienne Rifa, Christian Braegger, Annelies Geirnaert, Vanesa Natalin Rocha Martin, Christophe Lacroix

**Affiliations:** a Laboratory of Food Biotechnology, Institute of Food, Nutrition and Health, ETH Zurich, Zurich, Switzerland; b Division of Gastroenterology and Nutrition, University Children’s Hospital Zurich, Zurich, Switzerland; c Université Clermont Auvergne, INRA, UMRF, Aurillac, France; University of California, Davis

**Keywords:** *in vitro* model, infant gut microbiota, infantile colic, lactate-utilizing bacteria, pH, retention time

## Abstract

The metabolism of lactate impacts infant gut health and may lead to acute accumulation of lactate and/or H_2_ associated with pain and crying of colicky infants. Because gut microbiota studies are limited due to ethical and safety concerns, *in vitro* fermentation models were developed as powerful tools to assess effects of environmental conditions on the gut microbiota. In this study, we established a continuous colonic fermentation model (PolyFermS), inoculated with immobilized fecal microbiota and mimicking the proximal colon of 2-month-old infants. We investigated the effects of pH and retention time (RT) on lactate metabolism and of lactate-utilizing bacteria (LUB) exhibiting little or no H_2_ production. We observed that a drop in pH from 6.0 to 5.0 increased the number of lactate-producing bacteria (LPB) and decreased LUB concomitantly with lactate accumulation. Increasing RT from 5 to 10 h at pH 5.0 resulted in complete lactate consumption associated with increased LUB. Supplementation with dl-lactate (60 mM) to mimic lactate accumulation promoted propionate and butyrate production with no effect on acetate production. We further demonstrated that lactate-utilizing Propionibacterium avidum was able to colonize the reactors 4 days after spiking, suggesting its ability to compete with other lactate-utilizing bacteria producing H_2_. In conclusion, we showed that PolyFermS is a suitable model for mimicking young infant colonic microbiota. We report for the first time pH and RT as strong drivers for composition and metabolic activity of infant gut microbiota, especially for the metabolism of lactate, which is a key intermediate product for ecology and infant health.

**IMPORTANCE** The metabolism of lactate is important for infant gut health and may lead to acute lactate and/or H_2_ accumulation, pain, and crying as observed in colicky infants. Functional human studies often faced ethical challenges due to invasive medical procedures; thus, in this study, we implemented PolyFermS fermentation models to mimic the infant proximal colon, which were inoculated with immobilized fecal microbiota of two 2-month-old infants. We investigated the impact of pH, retention time, and accumulation of dl-lactate on microbiota composition and metabolic activity. We found that a drop in pH from 6.0 to 5.0 led to increased LPB and decreased LUB concomitantly with lactate accumulation. Increasing the RT resulted in complete lactate consumption associated with increased LUB. Our data highlight for the first time the impact of key abiotic factors on the metabolism of lactate, which is an important intermediate product for ecology and infant health.

## Abstract (precis)

The metabolism of lactate is important for infant gut health and may lead to acute lactate and/or H_2_ accumulation, pain, and crying as observed in colicky infants. Functional human studies often faced ethical challenges due to invasive medical procedures; thus, in this study, we implemented PolyFermS fermentation models to mimic the infant proximal colon, which were inoculated with immobilized fecal microbiota of two 2-month-old infants. We investigated the impact of pH, retention time, and accumulation of dl-lactate on microbiota composition and metabolic activity. We found that a drop in pH from 6.0 to 5.0 led to increased LPB and decreased LUB concomitantly with lactate accumulation. Increasing the RT resulted in complete lactate consumption associated with increased LUB. Our data highlight for the first time the impact of key abiotic factors on the metabolism of lactate, which is an important intermediate product for ecology and infant health.

## INTRODUCTION

The early establishment of gut microbes plays a crucial role in lifelong health and disease of infants (1). After birth, the infant gut becomes home to many microbes sourced from the mother and the environment. Primary colonizers are mainly lactate-producing bacteria (LPB) belonging to the genera *Bifidobacterium*, *Lactobacillus*, *Bacteroides*, *Streptococcus*, *Staphylococcus*, and *Enterococcus* (2–4). Hence, lactate is produced in large amounts from metabolism of dietary nondigestible (human milk oligosaccharides) and undigested (lactose) sugars and host mucins. Lactate is an important intermediate substrate for the gut microbiota that feeds lactate-utilizing bacteria (LUB). By combining detailed taxonomic (molecular) and functional (culture) assessment, we recently reported the importance of metabolic cross-feeding of lactate of the infant gut microbiota during the first 6 months of life and identified keystone species involved with lactate utilization (4). Moreover, lactate accumulation and metabolism were associated with infant gut health (5).

Infantile colic, or excessive crying of unknown cause, is a functional gastrointestinal disorder that affects up to 20% of infants (6, 7). In a recent study, colic phenotype was correlated positively with specific groups of *Proteobacteria*, including *Escherichia*, *Klebsiella*, *Serratia*, *Vibrio*, and *Pseudomonas*, but negatively with *Bacteroidetes* and *Firmicutes* phyla in the first weeks of life. A less diverse fecal microbiota was also observed in infants with colic (8). We recently reported specific LUB signatures for colicky and crying infants, supporting the hypothesis that increased H_2_ production by LUB could result in acute H_2_ accumulation, leading to pain and crying as observed for colicky infants (5). However, the huge interindividual variability of microbial composition poses a challenge to linking differences in the infant gut microbiota with health, symptoms, and disease. Moreover, sampling of the gut contents, particularly in the highly fermentative environment of the proximal colon, is highly restricted, especially for infants, due to ethical, accessibility, and safety concerns.

Alternatively, *in vitro* fermentation models were developed as powerful tools to study the effects of intrinsic and extrinsic factors on the composition and activity of human gut microbiota uncoupled from the host. Different systems, from simple anaerobic batch culture systems in flasks to multistage continuous flow models, have been developed to model fermentation in the colon, which harbors the highest density of microbes. However, colonic models should be carefully selected, with consideration given to their features and limits related to the scientific question addressed (9, 10). Inoculation of all fermentation models requires large amounts of fecal slurries, which is the most important limitation to infant colonic modeling. The composition, diversity, and function of infant gut microbiota in the first months of life largely differ from those of adults. The most profound differences are the elevated fecal lactate concentrations and the absence of or very low fecal butyrate levels, correlated with a low abundance of butyrate-producing bacteria (BPB) in infants, compared to the adult gut (4). Due to the rapid depletion of substrate and reduction of pH, which prevent further microbial activity, batch fermentation experiments are often restricted to short-term incubations. Continuous culture models are necessary to perform long-term studies under pseudo-steady-state conditions, with substrate replenishment and toxic product removal (10).

However, one of the main challenges of the continuous culture model is reproducing the high bacterial cell density and biofilm-associated microbes of the gut that are important to prevent washout of less-competitive bacteria. To address this, gut microbiota were immobilized in polysaccharide gel beads, starting from a small fecal inoculum volume, to mimic different hosts while maintaining high bacterial diversity and at cell densities in continuous intestinal reactors operating up to 120 days (11–15). Immobilized cell models mimicking the proximal colon of 4- to 8-month-old infants were successfully used to investigate the impact of retention time (RT) (16), prebiotics (17), and nucleosides and yeast extracts (15). To our knowledge, due to the technical difficulties of starting from small amounts of fecal samples, a fermentation model mimicking very young infant gut microbiota has not yet been implemented.

To gain insights into lactate metabolism, which plays an important role in infant gut health, we developed and validated for the first time a continuous fermentation model inoculated with immobilized fecal microbiota to mimic 2-month-old formula-fed infant gut microbiota. Using this model with the PolyFermS platform (12), we investigated the effect of important parameters (pH and RT) for lactate metabolism on the gut microbiota composition and activity. Furthermore, because lactate is an important intermediate product associated with infant colic, we investigated accumulation of lactate by supplementing dl-lactate and two infant lactate-utilizing bacterial strains (Propionibacterium avidum and Eubacterium limosum), selected for little or no H_2_ production, for their potential to colonize and metabolize residual lactate. A recent classification of the genus *Propionibacterium* allocated the cutaneous *P. avidum* to the new genus *Cutibacterium* (18).

## RESULTS

### Colonization of donor fecal microbiota in fermentation model.

**FIG 1:**
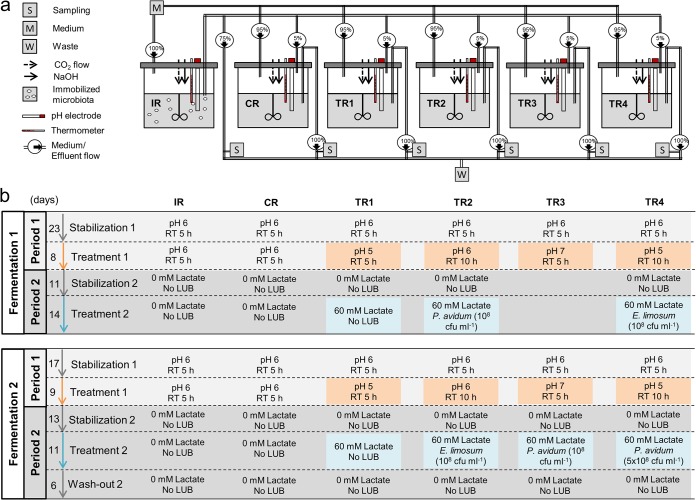
(a) Setup of the PolyFermS fermentation model inoculated with immobilized infant gut microbiota. The fermentation setup consisted of an inoculum reactor (IR) containing 30% (vol/vol) fecal beads, connected to a control reactor (CR) and four treatment reactors (TRs) continuously fed with 5% fermentation effluent from the IR and 95% fresh medium. All reactors were constantly flushed with CO_2_ to maintain anaerobiosis. Temperature was set at 37°C, stirring speed was set at 180 rpm, and pH was controlled automatically by the addition of 2.5 M NaOH. All reactors had a total working volume of 200 ml. (b) Setup of the experiment conditions at different periods. The fermentation of fecal samples from donor 1 (fermentation 1) and donor 2 (fermentation 2) was divided into 2 periods; each period consisted of stabilization and treatment and washout. RT, retention time; LUB, lactate-utilizing bacteria.

Two PolyFermS continuous fermentation models were used in this study to mimic the conditions in the proximal colon of a 2-month-old formula-fed infant. The fermentation setup consisted of a first inoculum reactor (IR) inoculated with 30% (vol/vol) gellan-xanthan gel beads that immobilized the fecal microbiota, which was connected to a control reactor (CR) and four treatment reactors (TRs). All TRs and the CR were operated in parallel, continuously inoculated with 5% fermentation effluent from the IR, and additionally fed with 95% fresh medium, as presented in Materials and Methods and illustrated in Fig. 1a. After an initial colonization and stabilization time of 11 days, the fermentations inoculated with fecal beads from donor 1 (fermentation F1) or donor 2 (fermentation F2) were divided into two experimental periods. Detailed experimental conditions for F1 and F2 with total times of 79 and 57 days, respectively, are depicted in Fig. 1b. During period 1, the effects of three levels of pH (5.0, 6.0, and 7.0) and two RTs (5 and 10 h) were studied. The effects of lactate supplementation (60 mM dl-lactate) and LUB strain addition on composition and activity of infant gut microbiota were investigated during period 2. Each period consisted of stabilization with CR control conditions for 11 to 23 days, followed by treatment application for 8 to 14 days.

Feces from two 2-month-old healthy infant donors with similar gestational ages, birth weights, ages, and feeding practices (see Table S1a in the supplemental material) were used for immobilization and model inoculation. The microbial composition of donor fecal samples was determined using qPCR targeting the 16S rRNA gene of specific characteristic bacterial groups for the infant gut microbiota (Table 1). The two selected donors harbored similar levels of *Firmicutes* and *Bacteroides*. Compared to donor 2, the fecal sample from donor 1 harbored higher levels of Enterobacteriaceae (9.4 and 7.9 log gene copies g^−1^, respectively), *Bifidobacterium* (9.2 and 7.6 log gene copies g^−1^), *Veillonella* (9.4 and 8.6 log gene copies g^−1^), and *Lactobacillus* (9.1 and 6.0 log gene copies g^−1^).

**TABLE 1:** 16S rRNA gene copy numbers of specific bacterial groups enumerated by qPCR^*a*^

Factor	Fermentation	Stabilization or treatment	Days	Log_10_ 16S rRNA gene copy no. of taxon ml^−1^ (mean ± SD):						
				*Firmicutes*	*Lactobacillus*	*Veillonella*	*Bacteroides*	*Bifidobacterium*	Enterobacteriaceae	*E. limosum*
Baseline in sample from										
Fecal donor^*b*^	1			9.88	9.10	9.35	9.34	9.24	9.39	ND
Inoculum reactor	1	Stabilization	21–23	10.01 ± 0.17	8.54 ± 0.34	8.81 ± 0.10	9.35 ± 0.14	9.45 ± 0.35	7.88 ± 0.17	ND
Control reactor	1	Stabilization	21–23	10.10 ± 0.10	8.22 ± 0.17	9.34 ± 0.30	9.45 ± 0.09	9.53 ± 0.26	8.58 ± 0.17	ND
Fecal donor^*b*^	2			9.68	6.00	8.64	9.46	7.56	7.85	2.68
Inoculum reactor	2	Stabilization	16–18	10.08 ± 0.14	6.71 ± 0.21	10.11 ± 0.31	9.58 ± 0.18	6.98 ± 0.30	8.01 ± 0.32	ND
Control reactor	2	Stabilization	16–18	10.28 ± 0.06	6.15 ± 0.10	10.88 ± 0.50	9.90 ± 0.05	7.13 ± 0.19	8.16 ± 0.08	ND
pH										
6.0	1	Stabilization	21–23	10.05 ± 0.08	7.82 ± 0.29	8.76 ± 0.22	9.44 ± 0.17	9.53 ± 0.27	8.15 ± 0.23	ND
5.0	1	Treatment	29–31	9.38 ± 0.12**	9.19 ± 0.08*	7.52 ± 0.08*	7.88 ± 0.10**	9 ± 0.07	8.68 ± 0.23**	ND
6.0	2	Stabilization	16–18	10.07 ± 0.16	6.19 ± 0.08	10.18 ± 0.16	9.58 ± 0.11	7.06 ± 0.25	7.87 ± 0.28	ND
5.0	2	Treatment	25–27	9.51 ± 0.03	7.85 ± 0.11**	8.68 ± 0.24	8.24 ± 0.14	8.06 ± 0.07	7.67 ± 0.04	ND
6.0	1	Stabilization	21–23	10.00 ± 0.14	8.03 ± 0.32	9.49 ± 0.49	9.46 ± 0.28	9.51 ± 0.23	8.61 ± 0.31	ND
7.0	1	Treatment	29–31	9.77 ± 0.05*	7.01 ± 0.11	9.35 ± 0.01	8.89 ± 0.03	8.32 ± 0.2*	8.83 ± 0.03	ND
6.0	2	Stabilization	16–18	10.01 ± 0.28	6.21 ± 0.06	10.26 ± 0.27	9.57 ± 0.15	7.21 ± 0.36	8.12 ± 0.07	ND
7.0	2	Treatment	25–27	10.09 ± 0.03	6.12 ± 0.11	10.39 ± 0.14	9.61 ± 0.05	7.05 ± 0.08	8.64 ± 0.12*	ND
RT (h)										
5	1	Stabilization	21–23	10.08 ± 0.02	7.68 ± 0.17	9.00 ± 0.08	9.4 ± 0.06	9.49 ± 0.2	8.49 ± 0.2	ND
10	1	Treatment	29–31	9.51 ± 0.05**	7.36 ± 0.32	8.47 ± 0.18*	9.27 ± 0.08	8.66 ± 0.04*	8.82 ± 0.19**	ND
5	2	Stabilization	16–18	10.14 ± 0.13	6.16 ± 0.13	10.35 ± 0.33	9.65 ± 0.18	7.28 ± 0.26	8.18 ± 0.16	ND
10	2	Treatment	25–27	10.06 ± 0.12	6.32 ± 0.27	10.10 ± 0.21	9.52 ± 0.23	7.68 ± 0.36	8.17 ± 0.21	ND
Supplemental lactate concn (mM)										
0	1	Stabilization	63–65	10.54 ± 0.26	8.94 ± 0.18	8.76 ± 0.17	9.85 ± 0.36	9.5 ± 0.07	8.22 ± 0.24	7.61 ± 0.35
60	1	Treatment	77–79	10.79 ± 0.26	8.13 ± 0.15*	8.94 ± 0.18	10.4 ± 0.59	9.57 ± 0.18	8.04 ± 0.40	8.02 ± 0.06
0	2	Stabilization	38–40	10.28 ± 0.09	7.64 ± 0.06	10.21 ± 0.33	9.45 ± 0.13	6.95 ± 0.24	8.58 ± 0.04	2.63 ± 0.10
60	2	Treatment	49–51	10.06 ± 0.27	7.61 ± 0.24	10.06 ± 0.37	9.04 ± 0.25	6.74 ± 0.19	8.58 ± 0.33	2.54 ± 0.61

a Data are means ± SD for the last 3 days of each stabilization and treatment; samples were analyzed in duplicate. Means with an asterisk (*) differ significantly between the previous stabilization and treatment within the same reactor within the same bacterial group: *, *P *<* *0.05; **, *P *<* *0.01; ND, not determined.

b Data for fecal donor are expressed as log_10_ CFU g^−1^ feces.

10.1128/mSystems.00264-18.10 TABLE S1 (a) Baseline characteristics of donors 1 and 2; (b) primers used for the detection of predominant bacterial groups in fecal and effluent samples by qPCR. Download Table S1, DOCX file, 0.02 MB. Copyright © 2019 Pham et al. 2019 Pham et al. This content is distributed under the terms of the Creative Commons Attribution 4.0 International license.

The IR and CR were operated under constant conditions at pH 6.0 with an RT of 5 h during the entire experiment and used for testing stability over 79 and 57 days in F1 and F2, respectively. After the first stabilization time that allowed gut microbiota to colonize the reactors and to reach steady conditions, the microbiota composition of the IR and CR detected by qPCR during both fermentations was very similar to the corresponding donor fecal sample for most of the targeted groups, including *Firmicutes*, *Bacteroides*, and *Bifidobacterium* (Table 1). Differences were observed for the levels of Enterobacteriaceae, which were 1.5 log lower in the IR (7.9 log gene copies ml^−1^) and 0.8 log lower in the CR (8.6 log gene copies ml^−1^) compared to the donor 1 fecal sample (9.4 log gene copies g^−1^). Also, 1.5- and 2.3-log-higher levels of *Veillonella* were detected in the IR (10.1 log gene copies ml^−1^) and CR (10.9 log gene copies ml^−1^) compared to the donor 2 fecal sample (8.6 log gene copies g^−1^), respectively.

### Fermentation stability.

To measure the metabolic and compositional stability of both fermentation models, we performed HPLC, qPCR, and MiSeq sequencing analyses of effluent samples of the IR and CR of F1 and F2. The metabolite ratios and concentrations for short-chain fatty acids (SCFAs; acetate, propionate, and butyrate), lactate, and formate measured with HPLC indicated overall stable microbial metabolic profiles in the IR and CR of both fermentations after an initial colonization and stabilization period of 17 days (Fig. S1). During F1, we observed an effect of time where the acetate concentration decreased (day 22, 128.8 mM; day 79, 94.7 mM) and the butyrate concentration increased (day 29, 8.1 mM; day 79, 21.6 mM), while the total C-mol concentration (mole of carbon per liter) calculated from addition of all metabolites remained stable (Fig. S1). These data suggest that the observed time drift in F1 is associated not with a loss of metabolic activity but instead with discrete equilibration of metabolism, with more acetate, as an intermediate metabolite, being converted into butyrate. Acetate was the main metabolite in effluents of both fermentations, followed by propionate and butyrate. While formate was not detected in the IR and CR of F1, it represented a significant fraction of approximately 20% (>20 mM) of the total metabolites of F2. The propionate concentration was lower, while the butyrate concentration was higher, in the IR and CR of F1 compared to F2. Furthermore, qPCR data showed stability of the bacterial groups of infant microbiota that were analyzed, including *Firmicutes*, Enterobacteriaceae, *Bacteroides*, *Bifidobacterium*, *Veillonella*, and *Lactobacillus* (Fig. S2). The model stability was confirmed by MiSeq data that showed an overall stable relative abundance of microbiota at the genus level in both reactors, with some fluctuations in the relative abundance of *Ruminococcus*, *Veillonella*, and *Prevotella* (Fig. S3).

10.1128/mSystems.00264-18.1 FIG S1 Lactate, formate, acetate, propionate, butyrate, and total metabolite concentration and total carbon number of the inoculum reactor (IR) and control reactor (CR) during fermentations 1 (a and b) and 2 (c and d). B, batch fermentation (see Materials and Methods). Download FIG S1, TIF file, 2.0 MB. Copyright © 2019 Pham et al. 2019 Pham et al. This content is distributed under the terms of the Creative Commons Attribution 4.0 International license.

10.1128/mSystems.00264-18.2 FIG S2 16S rRNA gene copy numbers (log_10_ copy numbers ml^−1^ fermentation effluent) of specific bacterial groups determined by qPCR in the inoculum reactor (IR) and control reactor (CR) during fermentations 1 (a and b) and 2 (c and d). Data were expressed as average from technical duplicates. Red, *Firmicutes*; black, Enterobacteriaceae; green, *Bacteroides*; purple, *Bifidobacterium*; blue, *Veillonella*; yellow, *Lactobacillus*. Download FIG S2, TIF file, 0.6 MB. Copyright © 2019 Pham et al. 2019 Pham et al. This content is distributed under the terms of the Creative Commons Attribution 4.0 International license.

10.1128/mSystems.00264-18.3 FIG S3 Relative abundance (%) of 16S rRNA genes at genus level analyzed in fermentation effluent using Illumina MiSeq V3-V4 amplicon sequencing in the inoculum reactor (IR) and control reactor (CR) during fermentations 1 (A) and 2 (B). Values <1% are summarized in the group “Other.” When assignment at genus level was not possible, the highest-level taxonomy assignment is shown. Download FIG S3, TIF file, 1.9 MB. Copyright © 2019 Pham et al. 2019 Pham et al. This content is distributed under the terms of the Creative Commons Attribution 4.0 International license.

No significant differences in composition and metabolic activity of the microbiota between the CR and TRs after the stabilization period were found in both fermentations (Fig. S4).

10.1128/mSystems.00264-18.4 FIG S4 Metabolic activity and composition of microbiota in the control reactor (CR) and treatment reactors (TR) at the end of stabilization periods. Values are mean results ± SD (*n* = 3). Download FIG S4, TIF file, 1.0 MB. Copyright © 2019 Pham et al. 2019 Pham et al. This content is distributed under the terms of the Creative Commons Attribution 4.0 International license.

In conclusion, qPCR detected similar levels of predominant groups for donor samples and the IR and CR. Differences in bacterial levels detected for the 2 donor fecal samples were well reproduced in the IR and CR of F1 and F2 and are reflected in distinct metabolic profiles. After an initial stabilization time of 17 days, we also demonstrated high stability of composition and metabolic activity of the microbiota over 79 and 57 days of continuous operation in the IR and CR operated with constant conditions for both F1 and F2, respectively.

### Impact of pH.

**FIG 2:**
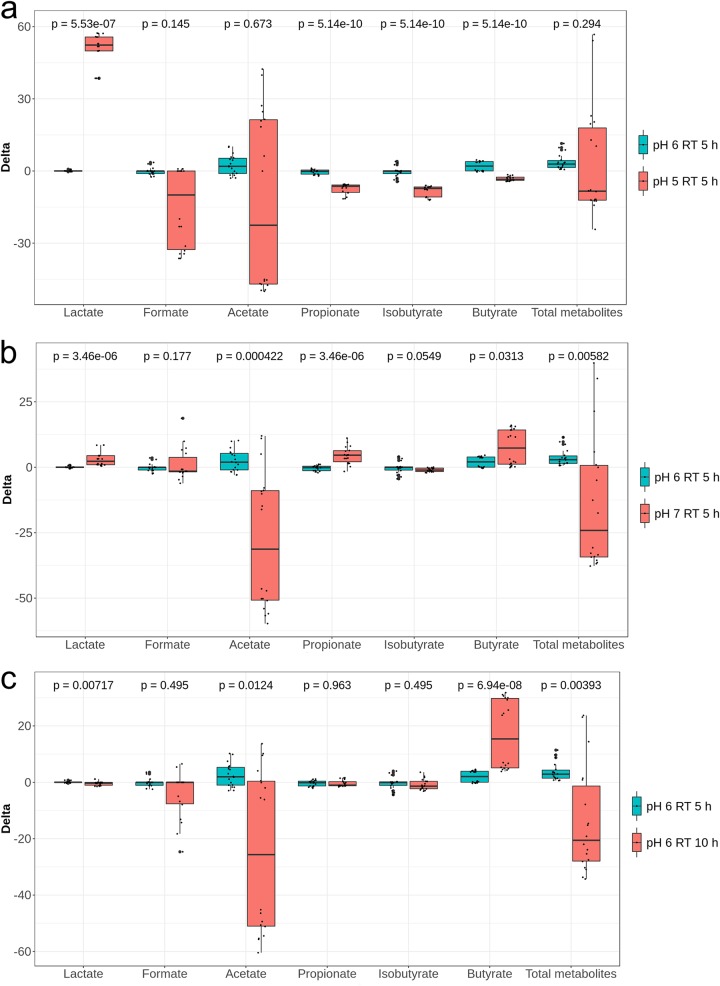
Effect of pH (a and b) and retention time (RT) (c) on the metabolic activity of infant gut microbiota using data from both fermentations. Values are expressed as differences (delta) of SCFA concentrations (mM) between treatment and previous stabilization period within each reactor.

**FIG 3:**
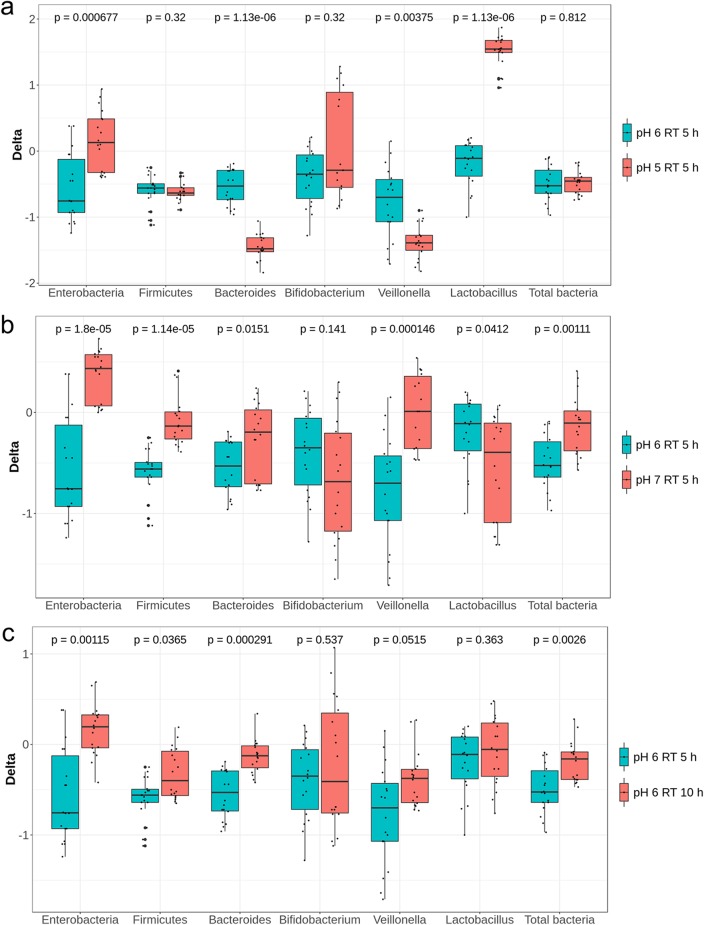
Effect of pH (a and b) and retention time (RT) (c) on levels of specific bacterial groups enumerated by qPCR using data from both fermentations. Values are expressed as differences (delta) of log_10_ 16S rRNA gene copy numbers of specific bacterial groups enumerated by qPCR between treatment and previous stabilization period within each reactor.

**FIG 4:**
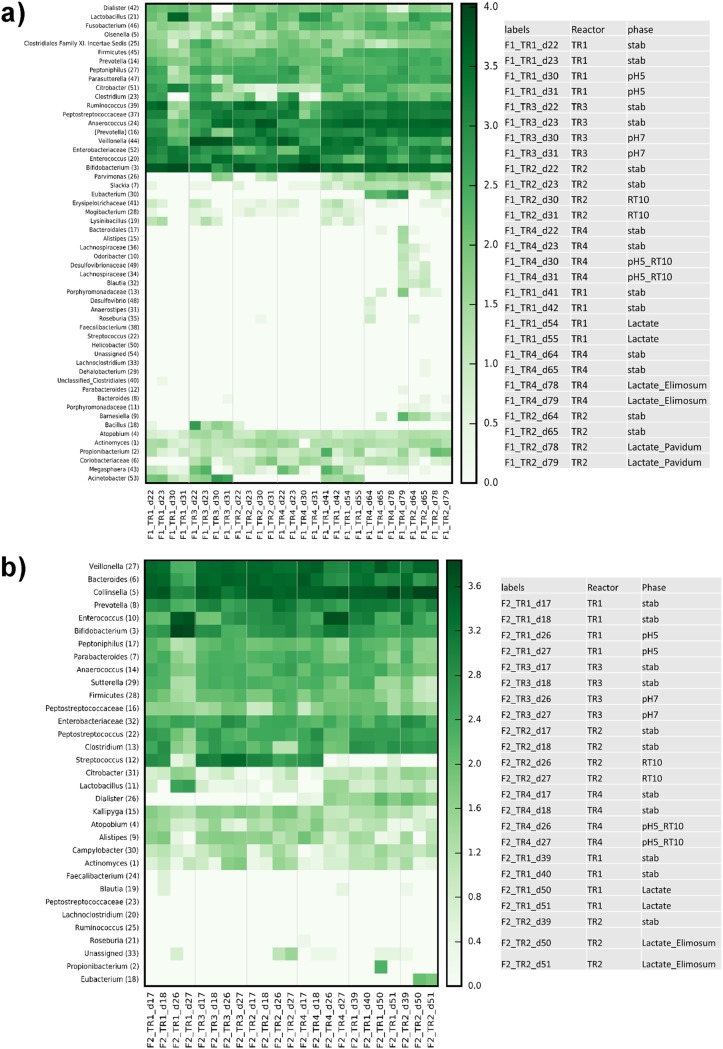
Impact of pH; retention time (RT) at pH 6.0; retention time (RT) at pH 5.0; and addition of lactate, *E. limosum*, and *P. avidum* on the relative abundance of 16S rRNA genes at genus level analyzed in fermentation effluent using Illumina MiSeq in fermentations 1 (a) and 2 (b). When assignment at genus level was not possible, the highest-level taxonomy assignment was shown.

During period 1 stabilization, all reactors were set at pH 6.0 and an RT of 5 h. Combinations of different pHs and RTs were then assigned to TRs during the following treatment period while CR conditions were kept constant (Fig. 1b). The pHs 5.0 and 7.0 were chosen to mimic the colonic pH of breast-fed (fecal pH 5.1 to 5.4 in the first 6 weeks) and formula-fed (fecal pH 7.0 to 8.2 from the second to the fifth week; fecal pH 6.4 after the fifth week) infants, respectively (19). For the statistical analysis of qPCR and metabolite data pooled from the two fermentations F1 and F2 inoculated with different microbiota, we calculated differences (delta) between treatment and previous stabilization period for each reactor. We compared the delta values of each treatment reactor (TR1 to TR4) with that of the control reactor (CR) measured during the same periods, using the nonparametric Wilcoxon rank sum test with false-discovery rate correction. Reducing the pH from 6.0 to 5.0 led to a significant increase in lactate (*P < *0.001), and decreases in propionate (*P* < 0.001), isobutyrate (*P < *0.001), and butyrate (*P < *0.001) production at pH 5.0 compared to pH 6.0 were shown (Fig. 2a). For both fermentations, significant lactate accumulation (from 0.6 ± 0.1 to 54.9 ± 3.9 mM in F1; from 0.0 to 47.7 ± 8.0 mM in F2; *P < *0.01) and significantly decreased propionate, butyrate, and isobutyrate (*P < *0.01 for F1 and *P < *0.05 for F2) production were measured at pH 5.0 compared to pH 6.0 (Fig. S5a and b). Moreover, a pH of 5.0 resulted in decreased acetate in F1 (*P < *0.001) or formate in F2 (*P < *0.05) relative to pH 6.0. Significantly lower levels of *Veillonella* (*P < *0.01) and *Bacteroides* (*P < *0.001) and higher levels of *Lactobacillus* (*P < *0.001) and Enterobacteriaceae (*P < *0.001) were measured using qPCR for effluent samples at pH 5.0 compared to pH 6.0 when combining data from the two fermentations (Fig. 3a) with fermentation (donor) effects (Table 1). Furthermore, lower relative abundances of *Veillonella* (F1, 1.5% versus 9.2%; F2, 1.2% versus 17.3%) and *Prevotella* (F1, 0.5% versus 5.7%; F2, 1.4% versus 5.4%) and higher relative abundances of *Lactobacillus* (F1, 22.2% versus 0.5%; F2, 2.8% versus 0.03%), *Enterococcus* (F1, 12.2% versus 4.4%; F2, 32.8% versus 1.0%), and *Bifidobacterium* (F1, 41.7% versus 30.5%; F2, 47.6% versus 3.5%) were recorded for both fermentations at pH 5.0 compared to pH 6.0 using MiSeq; however, no sequencing replicates prevent statistical analysis on MiSeq data (Fig. 4). During F1, low relative abundances of *Ruminococcus* (0.19% versus 19.3%) and *Peptostreptococcaceae* (0.19% versus 7.2%) and high relative abundances of *Citrobacter* (7.6% versus 2.8%) and Enterobacteriaceae (11.0% versus 6.6%) were measured at pH 5.0 compared to pH 6.0 (Fig. 4a). On the other hand, a strong decrease of the relative abundances of *Collinsella* (5.9% versus 29.9%) and *Bacteroides* (2.3% versus 21.4%) was observed in F2 at pH 5.0 compared to 6.0 (Fig. 4b).

10.1128/mSystems.00264-18.5 FIG S5 Effect of pH on the metabolic activity of infant gut microbiota in two PolyFermS models. (a and b) Concentrations of lactate, formate, and SCFA produced at pH 6.0 (black bar) and pH 5.0 (yellow bar) in fermentations 1 (a) and 2 (b). (c and d) Concentrations of lactate, formate, and SCFA produced at pH 6.0 (black bar) and pH 7.0 (yellow bar) in fermentations 1 (c) and 2 (d). Values are mean results ± SD (*n* = 3). *, *P < *0.05; **, *P < *0.01; ***, *P < *0.001. Download FIG S5, TIF file, 1.3 MB. Copyright © 2019 Pham et al. 2019 Pham et al. This content is distributed under the terms of the Creative Commons Attribution 4.0 International license.

Analysis of pooled data from the two fermentations showed a significant decrease in acetate (*P < *0.001) and an increase in butyrate (*P < *0.05) (Fig. 2b) and a significant increase in Enterobacteriaceae (*P < *0.001), *Firmicutes* (*P < *0.001), *Veillonella* (*P < *0.001), *Bacteroides* (*P < *0.05), and total bacteria (*P < *0.01) at pH 7.0 compared to pH 6.0 (Fig. 3b). The impact of the high pH of 7.0 (TR3) on microbial composition and metabolic activity was fermentation (donor) dependent (Table 1; Fig. S5c and d). No significant effect of pH 7.0 on either microbial composition or metabolic activity was found in F2 compared to pH 6.0. In contrast, during F1, pH 7.0 significantly decreased acetate production (*P < *0.01) and *Firmicutes* and *Bifidobacterium* levels (*P < *0.05) and increased butyrate (*P < *0.01) and formate (*P < *0.05) accumulation compared to pH 6.0. Lower relative abundances of *Bifidobacterium* and *Prevotella* and higher relative abundances of *Enterococcus* were also observed in both fermentations at pH 7.0 compared to 6.0 (Fig. 4a and b). Furthermore, the relative abundance of *Anaerococcus* increased and that of *Veillonella* decreased during F1, while *Bacteroides* and *Streptococcus* increased and *Collinsella* decreased during F2, at pH 7.0 compared to pH 6.0, although no statistical testing could be done.

### Impact of retention time.

The effect of RT (5 and 10 h) on the gut microbiota composition and metabolic activity was tested at pH 6.0 in TR2 of both models during experimental period 1 (Fig. 1b). An RT of 10 h significantly increased butyrate production (*P < *0.001) compared to 5 h with pooled data from both fermentations (Fig. 2c) and by 4-fold and 2.5-fold in F1 and F2, respectively (Fig. S6a and b). A longer RT also led to significantly lower acetate in F1 (*P < *0.05) and total metabolite (*P < *0.01) levels compared to an RT of 5 h (Fig. S6a). A 10-h RT significantly increased total bacteria (*P < *0.01), *Firmicutes* (*P < *0.05), Enterobacteriaceae (*P < *0.01), and *Bacteroides* (*P < *0.001) when pooling data from the two fermentation (Fig. 3c). We also measured decreased *Bifidobacterium* (*P < *0.05) and *Veillonella* (*P < *0.05) in F1 compared to those with a 5-h RT (Table 1). In contrast, no impact of RT was found for the microbial composition of F2 using qPCR. However, decreased *Bifidobacterium* and increased Enterobacteriaceae abundances during F1 and at 10-h RT compared to 5-h RT were confirmed by MiSeq data (Fig. 4a and b). Furthermore, lower *Ruminococcus* and higher *Anaerococcus* abundance during F1, lower *Streptococcus* abundance during F2, and higher *Prevotella* abundance during both fermentations were observed at the 10-h RT than at the 5-h RT (Fig. 4a and b).

10.1128/mSystems.00264-18.6 FIG S6 Effect of retention time (RT) on the metabolic activity of infant gut microbiota in two PolyFermS models. Concentrations of lactate, formate, and SCFA produced at 5-h (black bar) and 10-h (yellow bar) RTs at pH 6.0 (a and b) and at pH 5.0 (c and d). Values are mean results ± SD (*n* = 3). *, *P < *0.05; **, *P < *0.01; ***, *P < *0.001. Download FIG S6, TIF file, 1.3 MB. Copyright © 2019 Pham et al. 2019 Pham et al. This content is distributed under the terms of the Creative Commons Attribution 4.0 International license.

We also compared TR1 (pH 5.0; RT, 5 h) and TR4 (pH 5.0; RT, 10 h) during period 1, because similar conditions were used for all TRs during the stabilization period, resulting in similar microbiota composition and activities (Fig. S4). At pH 5.0, a 10-h RT significantly decreased lactate accumulation compared to a 5-h RT (*P < *0.001) in both fermentations (Fig. S6c and d). MiSeq data showed a trend for higher relative abundance of lactate-producing *Enterococcus* in both fermentations at 10-h RT compared to 5-h RT. Moreover, a lower abundance of *Lactobacillus* and higher abundances of *Bifidobacterium*, *Enterococcus*, and *Anaerococcus* during F1 were observed at 10-h RT compared to 5-h RT at pH 5.0 (Fig. 4b). In contrast, a lower abundance of *Bifidobacterium* and a higher abundance of *Collinsella* and *Veillonella* were observed during F2 at 10-h RT compared to 5-h RT at pH 5.0 (Fig. 4b).

### Impact of dl-lactate supplementation.

**FIG 5:**
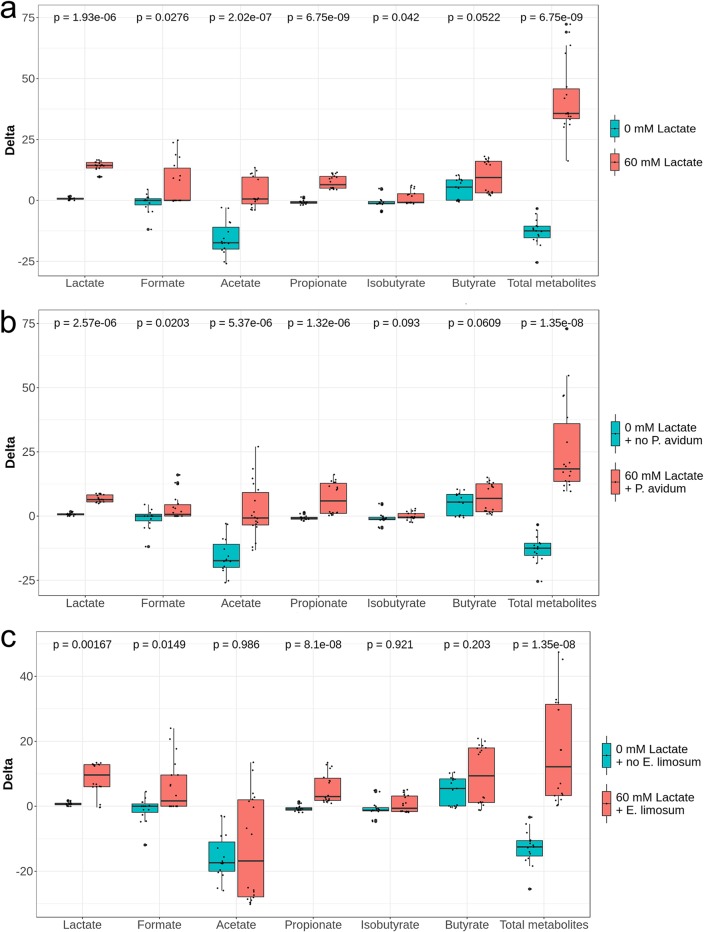
Effect of the addition of lactate (a) and of lactate and lactate-utilizing bacteria (b and c) on the metabolic activity of infant gut microbiota using data from both fermentations. Values are expressed as differences (delta) of SCFA concentrations (mM) between treatment and previous stabilization period within each reactor.

**FIG 6:**
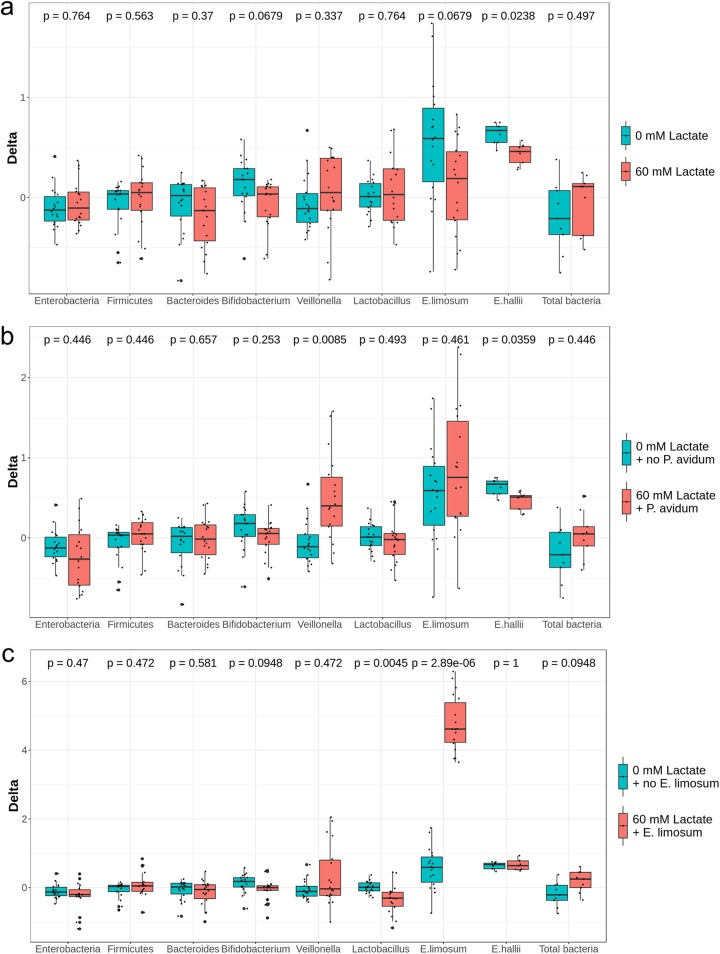
Effect of the addition of lactate (a) and of lactate and lactate together with lactate-utilizing bacteria (b and c) on levels of specific bacterial groups enumerated by qPCR using data from both fermentations. Values are expressed as differences (delta) of log_10_ 16S rRNA gene copy numbers of specific bacterial groups enumerated by qPCR between treatment and previous stabilization period within each reactor.

Supplementation with 60 mM dl-lactate in nutritive medium to mimic the accumulation of lactate in the infant gut resulted in significant lactate accumulation (*P < *0.001) as well as an increase in acetate (*P < *0.001), propionate (*P < *0.001), and total SCFA (*P < *0.001) production compared to no supplementation, when combining data from the two fermentations (Fig. 5a). Lactate accumulations in the effluent were similar, of 11.7 ± 1.9 mM and 12.8 ± 2.7 mM for F1 and F2, respectively. Significant fermentation (donor)-dependent increases in propionate and butyrate were detected (Fig. S7a and b). l-Lactate determination by enzymatic assay revealed the presence of both d- and l-isomers of lactate in reactors supplemented with 60 mM dl-lactate in F1 (47.9 and 52.1%, respectively) and F2 (71.5 and 28.5%). No significant effect of lactate supplementation on microbial composition by qPCR was observed (Table 1 and Fig. 6a), except for a small but significant decrease of Eubacterium hallii (*P < *0.05) with addition of lactate. In contrast, adding 60 mM dl-lactate appeared to affect microbial relative abundances of some groups, with observed increased *Peptostreptococcaceae* (10.9% versus 7.1%) and decreased *Citrobacter* (0.6% versus 3.9%) and Enterobacteriaceae (4.2% versus 11.3%) abundances in F1, and increased *Collinsella* (43.4% versus 29.3%) and *Veillonella* (22.8% versus 14.0%) and decreased *Bacteroides* (5.7% versus 14.1%) and *Enterococcus* (1.5% versus 8.8%) abundances in F2 (Fig. 4).

10.1128/mSystems.00264-18.7 FIG S7 Effect of the addition of lactate and lactate-utilizing bacteria on the metabolic activity of infant gut microbiota in two PolyFermS models. Concentrations of lactate, formate, and SCFAs produced without (black bar) and with addition of 60 mM dl-lactate (yellow bar) in F1 (a) and F2 (b); without (black bar) and with addition of 60 mM dl-lactate and 10^8^ CFU ml^−1^ *E. limosum* (yellow bar) in F1 (c) and F2 (b); and without (black bar) and with addition of 60 mM dl-lactate and 10^8^ CFU ml^−1^ *P. avidum* (yellow bar) in F1 (e) and F2 (f). Values are mean results ± SD (*n* = 3). *, *P < *0.05; **, *P < *0.01; ***, *P < *0.001. Download FIG S7, TIF file, 0.5 MB. Copyright © 2019 Pham et al. 2019 Pham et al. This content is distributed under the terms of the Creative Commons Attribution 4.0 International license.

### Impact of addition of lactate-utilizing bacteria with dl-lactate supplementation.

*P. avidum* or *E. limosum* was selected among infant LUB for its capacity to utilize lactate with no or little H_2_ production, respectively. We tested the impact of daily spiking with each strain individually at a high cell concentration (10^8^ CFU ml^−1^) in reactors supplemented with 60 mM dl-lactate to mimic lactate accumulation.

*E. limosum* was detected at high levels in F1 and F2 (8.9 ± 0.2 and 7.8 ± 0.4 log copies ml^−1^, respectively) in reactors 22 h after spiking (Table 2 and Fig. 6c). When pooling data from the two fermentations, *E. limosum* and lactate supplementation led to significant increases in lactate (*P < *0.01), formate (*P < *0.05), propionate (*P < *0.001), and total SCFAs (*P < *0.001) (Fig. 5c). The impact of daily addition of *E. limosum* at 10^8^ CFU ml^−1^ together with 60 mM dl-lactate differed between fermentations (Fig. S7c and d). *E. limosum* and lactate supplementation significantly increased butyrate (30.3 ± 1.3 mM versus 18.7 ± 1.2 mM, *P < *0.01) in F1 and propionate (20.6 ± 2.5 mM versus 11.2 ± 2.9, *P < *0.05) in F2 compared to respective stabilization. This treatment also decreased Enterobacteriaceae detected by qPCR in F1 (*P < *0.05) (Table 2), which was confirmed by MiSeq data in both fermentations. A lower abundance of *Enterococcus* was also observed in both fermentations, with a higher relative abundance of *Bifidobacterium*, *Peptostreptococcaceae*, and *Ruminococcus* during F1 and of *Collinsella* and lactate-utilizing *Veillonella* during F2 and a lower *Bacteroides* abundance during F2 (Fig. 4).

**TABLE 2:** Effect of supplementation with dl-lactate and *E. limosum* or *P. avidum* on specific bacterial groups enumerated by qPCR^*a*^

Lactate (mM) plus organism	Fermentation	Stabilization or treatment	Days	Log_10_ 16S rRNA gene copy no. of taxon ml^−1^ (mean ± SD):						
				Enterobacteriaceae	*Firmicutes*	*Bacteroides*	*Bifidobacterium*	*Veillonella*	*Lactobacillus*	*E. limosum*
0	1	Stabilization	63–65	9.00 ± 0.04	10.41 ± 0.09	9.50 ± 0.35	9.50 ± 0.14	8.23 ± 0.17	8.38 ± 0.28	8.30 ± 0.07
60 + *E. limosum*	1	Treatment	77–79	8.27 ± 0.13*	10.58 ± 0.54	10.11 ± 1.06	9.54 ± 0.17	8.30 ± 0.42	7.55 ± 0.29	8.91 ± 0.18*
0	2	Stabilization	38–40	9.04 ± 0.17	10.05 ± 0.41	9.36 ± 0.32	6.87 ± 0.49	9.89 ± 0.62	7.81 ± 0.39	2.51 ± 0.76
60 + *E. limosum*	2	Treatment	49–51	8.76 ± 0.67	10.23 ± 0.40	9.03 ± 0.42	6.75 ± 0.23	10.75 ± 1.02	7.52 ± 0.50	7.82 ± 0.41*
0	1	Stabilization	63–65	8.93 ± 0.12	10.35 ± 0.10	9.36 ± 0.12	9.46 ± 0.03	8.00 ± 0.02	8.93 ± 0.19	5.99 ± 0.39
60 + *P. avidum*	1	Treatment	77–79	8.44 ± 0.28*	10.73 ± 0.31	10.07 ± 1.00	9.62 ± 0.09	8.07 ± 0.35	8.51 ± 0.29	7.4 ± 0.07*
0	2	Stabilization	38–40	8.59 ± 0.31	10.09 ± 0.24	9.35 ± 0.11	6.82 ± 0.25	9.78 ± 0.22	7.81 ± 0.16	2.33 ± 0.40
60 + *P. avidum*	2	Treatment	49–51	8.61 ± 0.21	10.00 ± 0.16	9.17 ± 0.20	6.73 ± 0.21	10.48 ± 0.63	7.57 ± 0.10	3.42 ± 1.16

a dl-Lactate and *E. limosum* were supplemented at 60 mM ml^−1^, and *P. avidum* was supplemented at 10^8^ CFU ml^−1^. Means with an asterisk (*) differ significantly between the previous stabilization and treatment within the same reactor within the same bacterial group: *, *P* < 0.05.

Due to the lack of specific primers for *Propionibacterium*, we quantified *P. avidum* by specific plating of effluent samples of all reactors during stabilization, after daily spiking with 1 × 10^8^ (TR3) or 5 × 10^8^ (TR4) CFU ml^−1^ *P. avidum* and during the washout period after the treatment during F2 (Fig. S8a). Before treatment, *P. avidum* viable cell counts were similar and low in all reactors (4.1 ± 0.4 log CFU ml^−1^). *P. avidum* reached similar high cell counts of 8.6 ± 0.9 log CFU ml^−1^ (TR3) and 8.4 ± 0.1 log CFU ml^−1^ (TR4) for the two addition levels. Interestingly, 4 days after the last *P. avidum* addition, significantly higher levels were detected in TR3 and TR4 by plate counts (6.7 ± 0.3 and 5.9 ± 0.4 CFU ml^−1^, respectively) compared to nontreated reactors (TR1, 4.8 ± 0.1 CFU ml^−1^; TR2, 4.0 ± 0.1 CFU ml^−1^) (*P < *0.001; except TR4 versus TR1, *P < *0.05).

10.1128/mSystems.00264-18.8 FIG S8 (a) Viable counts (log_10_ CFU ml^−1^ fermentation effluent) of *P. avidum* measured by plate counts of reactor effluents before, during, and after supplementation with *P. avidum* in F2. Yellow and orange bars indicate reactors that were spiked with 10^8^ CFU ml^−1^ (TR3) and 5 × 10^8^ CFU ml^−1^ (TR4) *P. avidum*, respectively, during treatment. Values are mean results ± SD (*n* = 3). (b) *P. avidum* in reactor effluents compared to theoretical washout curves before, during, and after supplementation with 10^8^ CFU ml^−1^ (left) and 5 × 10^8^ CFU ml^−1^ (right) *P. avidum* in F2. Download FIG S8, TIF file, 0.3 MB. Copyright © 2019 Pham et al. 2019 Pham et al. This content is distributed under the terms of the Creative Commons Attribution 4.0 International license.

The addition of *P. avidum* at 10^8^ CFU ml^−1^ together with 60 mM dl-lactate resulted in a significant increase of lactate (*P < *0.001), acetate (*P < *0.001), propionate (*P < *0.001), and total metabolite (*P < *0.001) production when combining data from the two fermentations (Fig. 5b). In F1, this treatment also led to increased butyrate concentration (*P < *0.001), decreased Enterobacteriaceae (0.5 log copy number), and increased *E. limosum* (1.5 log copy number) (Table 2 and Fig. S7e). Combining data from the two fermentations indicated significant increase in *Veillonella* (*P < *0.01) and decrease in *E. hallii* (*P < *0.05) levels after the addition of *P. avidum* and lactate compared to stabilization with this treatment (Fig. 6b). Trends toward higher *Anaerococcus* and *Ruminococcus* and lower *Enterococcus* abundances were also observed during F1 (Fig. 4).

**FIG 7:**
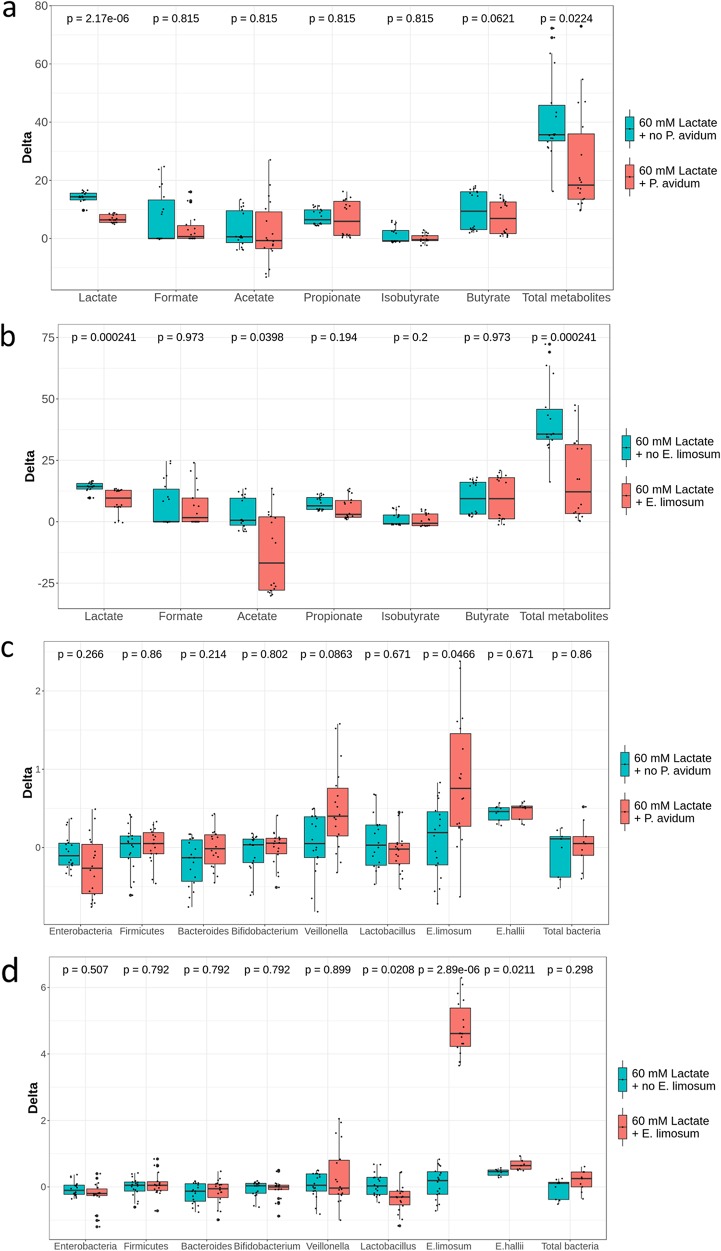
Effect of the addition of lactate-utilizing bacteria on the metabolic activity of infant gut microbiota (a and b) and on levels of specific bacterial groups enumerated by qPCR (c and d) using data from both fermentations. Values are expressed as differences (delta) of SCFA concentrations (mM) and log_10_ 16S rRNA gene copy numbers of specific bacterial groups enumerated by qPCR between treatment and previous stabilization period within each reactor.

Compared to 60 mM dl-lactate, the addition of 10^8^ CFU ml^−1^ *P. avidum* together with 60 mM dl-lactate led to a decrease in lactate (*P < *0.001) and an increase in *E. limosum* (*P < *0.05) (Fig. 7a and c). Addition of 10^8^ CFU ml^−1^ *E. limosum* together with 60 mM dl-lactate decreased lactate (*P < *0.001) and acetate (*P < *0.05) and increased levels of *E. limosum* (*P < *0.001) and *E. hallii* (*P < *0.05) compared to 60 mM dl-lactate alone (Fig. 7d).

To further demonstrate the impact of treatments on the infant PolyFermS microbiota of donors 1 and 2, we performed principal-coordinate analysis (PCoA) of weighted and unweighted UniFrac distance (Fig. S9). In fermentation 2, PCoA showed a clear separation of the treated microbiota from the untreated control, whereas this separation was less clear in fermentation 1.

10.1128/mSystems.00264-18.9 FIG S9 PCoA of weighted and unweighted UniFrac distance of treated and nontreated microbiota in control and treatment reactors in fermentations 1 and 2. Red, CR and TRs during no treatment. Other colors, TRs during treatment; purple, pH 5; turquoise, pH 7; yellow, pH 5 plus 10-h RT; pink, 10-h RT; blue, lactate; green, lactate plus *P. avidum*; orange, lactate plus *E. limosum*. Download FIG S9, TIF file, 1.0 MB. Copyright © 2019 Pham et al. 2019 Pham et al. This content is distributed under the terms of the Creative Commons Attribution 4.0 International license.

## DISCUSSION

### PolyFermS closely mimics the young infant gut microbiota.

The initial colonization of the gut is important for both short- and long-term health of infants (3). Infant gut microbiota studies using 16S rRNA-based analysis of fecal samples have provided crucial data on the composition and diversity of the gut microbiota and the effects of many factors, such as delivery mode (20) and diet (21). However, molecular methods can provide only limited insights into mechanisms and functions of bacterial species. Moreover, functional *in vivo* studies in humans often face social and ethical challenges due to invasive medical procedures (9, 10). In this study, for the first time we reported gut fermentation models to mimic the proximal colon of a 2-month-old infant and investigated the impact of abiotic and biotic factors to modulate infant gut microbiota composition and metabolic activity.

Large individual variations in gut microbiota composition and diversity in the first months of life have been well demonstrated in recent studies (2, 4, 21). The two infant donors used to inoculate the PolyFermS models harbored very different microbial compositions and *in vitro* metabolic profiles. The levels of Enterobacteriaceae, *Bacteroides*, *Bifidobacterium*, *Lactobacillus*, and total bacteria of the two infant donors were within the ranges reported in previous publications (17, 22–24). Distinct microbial compositions of fecal inoculum samples were reflected in different microbial compositions and metabolic activities of the microbiota during fermentations, such as high propionate-producing *Veillonella* levels together with high propionate production for donor 2 and in the IR and CR of F2, compared to donor 1 and F1.

The levels of predominant bacterial groups detected by qPCR in the IR and CR, with the exception of *Veillonella*, were similar to the corresponding donor fecal samples, suggesting that the gut microbiota from donor fecal samples were well conserved during sampling, immobilization, and cultivation under the conditions selected for the formula-fed young infant model. The preparation of the bead inoculum used only small amounts of fecal microbiota which could be obtained from ca. only 1 g of fecal material for production of approximately 200 ml of beads, and only 60 ml of beads was required for inoculation of the IR. This, with the reproduction of both the planktonic and sessile microbiota of the colon, is a unique feature of immobilization and using PolyFermS for modeling young, and possibly preterm, infant gut microbiota when only very limited volumes of feces are available. High and stable microbial concentrations, and stable relative abundances comparable to the fecal sample, were measured in the IR and CR throughout the 79- and 57-day fermentations. These data, combined with SCFA data, indicate long-term stability of fermentation models inoculated with infant fecal beads. PolyFermS models can be expanded to various configurations, allowing comparison of treatments and a control with the same microbiota (12, 13, 15, 25). In this study, the PolyFermS model, which combines four treatments with a control reactor operated with constant conditions and inoculated with identical microbiota as produced in the IR, appears well suited for testing a range of abiotic and biotic factors of infant gut fermentation and requires only a minimal amount of fecal material for inoculation.

### Low pH increased LPB and decreased LUB concomitantly with lactate accumulation.

*In vitro* fermentations with fecal inocula from 6-month-old infants, children, and adults have demonstrated the impact of environmental conditions, such as pH and RT, on the gut microbiota composition and lactate metabolism (12, 16, 26, 27). Little is known about the impact of such factors on the gut microbiota of younger infants, mainly because suitable gut fermentation models were lacking. Furthermore, lactate is one of the most important intermediate metabolites in the infant gut, and its accumulation can be detrimental for health (28). Using PolyFermS models, we investigated the impact of colonic pH and RT, which are known to vary widely in infants, and simulated lactate accumulation to determine the impact on 2-month-old infant gut microbiota and lactate metabolism.

The colonic pH can have a profound effect on the composition and metabolic activity of the human gut microbiota. A study investigating the effects of pH (5.2, 5.9, and 6.4) on lactate production and utilization in batch cultures inoculated with fecal slurries from four adult donors showed that pH 5.2 induced lactate accumulation due to reduced utilization (27). Using a single-stage continuous model inoculated with immobilized 6-month-old infant fecal microbiota, Cinquin et al. reported that the proportion of lactate significantly decreased when both the pH and RT were increased simultaneously, mimicking conditions from proximal to distal colon (16). Lactate utilization plays a central role in the metabolism of infant gut microbiota and could have a direct impact on infant health (4, 5, 29). To our knowledge, this is the first study investigating the impact of pH on infant gut microbiota composition and metabolic activity using *in vitro* colonic fermentation models. The selection of pH 5.0, 6.0, and 7.0 in this study was physiologically relevant, considering that infant stool pH varies from 4.8 to 7.0 in the first month of life (30). A recent study investigating the effect of Bifidobacterium infantis supplementation on fecal pH showed that the mean fecal pH of the probiotic group was 5.15, whereas the control group had a fecal pH of 5.97 (31).

One important finding in this study is the effect of low pH on fermentation under conditions mimicking the infant proximal colon. A low pH of 5.0 led to lactate accumulation and significantly decreased propionate and butyrate production, which agrees with data in adults (27). The decrease of propionate levels at pH 5.0 compared to pH 6.0 could be explained by a lower abundance of *Veillonella* bacteria, which are the main producers of propionate in the infant gut (4). Similarly, the decrease in butyrate production at pH 5.0 may be associated with lower abundance of butyrate-producing *Anaerococcus*. The accumulation of lactate at pH 5.0 agrees with the observed higher abundance of LPB (i.e., *Lactobacillus*, *Enterococcus*, and *Bifidobacterium*) and lower abundance of LUB (i.e., *Veillonella*). Consistent with previous studies (12, 26, 32), we also observed an inhibition of *Bacteroides* by acidic pH, as shown by both qPCR and MiSeq analyses.

### Increasing RT resulted in complete lactate consumption at low pH, associated with increased LUB.

Formula-fed infants showed a large variation in gastrointestinal transit time, with mean RTs of 13.7 h (range, 7.1 to 35.2 h) and 17.4 h (range, 5.4 to 36.5 h) at age 17 and 113 days, respectively (33), while the proximal colon transit time is estimated to be about one-third of the total transit time. In this study, we demonstrated that proximal colonic transit time is a strong determinant of the 2-month-old infant gut microbiota composition and metabolism *in vitro*. We showed that the effect of RT is pH and donor dependent. Increasing RT from 5 to 10 h at pH 5.0 attenuated the effect of low pH on the gut microbiota composition and metabolic activity and reduced lactate accumulation. This effect could be explained by the lower abundance of lactate-producing *Lactobacillus* and the higher abundance of lactate-utilizing *Veillonella* upon increased RT. We suggested that increased RT promotes the establishment of the trophic chain and the reutilization of lactate. In agreement, a recent study using an *in vitro* continuous fermentation system inoculated with adult fecal microbiota also reported that the abundance of *Veillonellaceae* (including genus *Veillonella*) increased with prolonged RT (34). Increasing RT from 5 to 10 h at pH 5.0 resulted in a small but significant increase of isobutyrate, suggesting an elevation of proteolytic activity possibly due to carbohydrate limitations (35).

At pH 6.0, a 10-h RT led to a lower abundance of *Bifidobacterium* and a higher abundance of Enterobacteriaceae relative to a 5-h RT. This observation agrees with previous studies that showed that *Bifidobacterium* spp. were less abundant in feces from functional constipated adult patients (36) and that Enterobacteriaceae levels were higher and *Bifidobacterium* levels were lower in constipated-irritable bowel syndrome (IBS) adults (C-IBS) compared to healthy adults (37). Increasing RT at pH 6.0 favored butyrate production in both fermentations concomitantly with a decrease of the intermediate products acetate (F1) and formate (F2). This observation could be explained by the slow kinetics and low levels of butyrate producers in the infant microbiota, which cannot efficiently reuse intermediate products such as lactate, succinate, and acetate when the RT is short. Our data provide initial mechanistic insights into the possible impact of transit time on infant gut microbiota composition and activity.

### Supplementation with lactate and LUB reduced Enterobacteriaceae and increased SCFAs.

Because most primary colonizers in the infant gut are LPB, lactate must be efficiently reused to prevent negative consequences of lactate accumulation. However, excess H_2_ production from lactate utilization (e.g., by *Veillonella*) may also lead to flatulence and is a possible factor in infantile colic (38). Indeed, we recently reported higher lactate-utilizing, H_2_-producing bacteria in colicky infants (5). On the other hand, LUB that produce only minimal or no H_2_ (e.g., *E. limosum* and *P. avidum*) were shown to compete with high H_2_-producing LUB (e.g., *Veillonella*) in pure and mixed cultures using anaerobic techniques (5).

In this study, a large amount of lactate (ca. 80% of 60 mM added dl-lactate) was reused, confirming the efficient utilization of lactate by LUB. Furthermore, adding 60 mM dl-lactate to mimic lactate accumulation increased butyrate and propionate formation. Interestingly, the impact of lactate was detected only on a functional but not on a taxonomic level, suggesting that lactate increased the activity of LUB by providing more energetic substrate but not by stimulating growth to detectable levels. Infant LPB, including *Lactobacillus*, produce both d- and l-lactate. The two isomers of lactate were detected at comparable levels after the addition of 60 mM dl-lactate, suggesting that the 2-month-old infant LUB community was able to utilize both d- and l- forms. Our data suggest that LUB of infant colonic microbiota have a high capacity to metabolize lactate, possibly as a natural protective mechanism in infant microbiota preventing lactate accumulation and detrimental health effects such as acidosis.

The *E. limosum* and *P. avidum* strains tested in this study were isolated from healthy infant feces and characterized for their ability to metabolize different substrates (5). While *E. limosum* utilizes lactate to produce butyrate, *P. avidum* produces propionate, acetate, and CO_2_. Lyophilized *E. limosum* fed to mice significantly attenuated colitis and increased cecal butyrate levels compared to the control group (5, 39). In our study, *E. limosum*, combined with the supplementation with 60 mM dl-lactate, led to a lower relative abundance of the Enterobacteriaceae family. The treatment also promoted acetate and butyrate production in F1 and propionate in F2, consistently with the butyrogenic and propionigenic profiles of donors 1 and 2, respectively. The increase of propionate might be attributed to the addition of lactate, which further stimulates the lactate-utilizing propionate-producing bacteria. The increase of butyrate and propionate may be of clinical significance for the infant gut, because of their well-established beneficial impacts on host health. Butyrate is the main energy source for enterocytes and regulates the epithelial barrier and immunity functions of the epithelial cells (40, 41). Furthermore, butyrate has been implicated in protection against colitis and colorectal cancer (42). On the other hand, propionate has been shown to stimulate an anti-inflammatory response (43).

*Propionibacterium*, recently reclassified in two different genera, *Propionibacterium* and *Cutibacterium* according to dairy and skin origin, respectively, is one of the dominant organisms of the skin microbiota (44). Recent studies have reported its natural occurrence in breast milk (45, 46), as well as in neonatal feces (47, 48). The addition of *P. avidum* with 60 mM dl-lactate increased concentrations of both lactate and the main SCFAs, decreased Enterobacteriaceae, and increased butyrate-producing *E. limosum* by 1.5 log. The increase of butyrate could be explained by the increase of *E. limosum*. Furthermore, *P. avidum* produces acetate, which could be used by butyrate producers. Moreover, in comparison with the theoretical washout curves of *P. avidum* spiked at 1 × 10^8^ and 5 × 10^8^ CFU ml^−1^, calculated for a 5-h RT in a homogenous continuous stirred-tank reactor (see Fig. S8b in the supplemental material), our data demonstrated the ability of *P. avidum* to colonize the reactors 4 days after spiking.

In conclusion, we successfully implemented for the first time stable continuous colonic fermentation models to mimic the proximal colon of very young infants using immobilized fecal microbiota. Using the PolyFermS model platform, we observed a strong impact of pH and RT on the composition and metabolic activity of the gut microbiota involved in lactate metabolism, which is important for ecology and infant health. Using two different donors with different microbiota reflects the *in vivo* situation, where interindividual variability is inevitable and unavoidable and further strengthens the impacts detected in both fermentations.

## MATERIALS AND METHODS

### Bacterial strains and growth conditions.

*P. avidum* (strain 4118; Laboratory of Food Biotechnology, ETH Zurich) was previously isolated from feces of a healthy infant (5). The strain was activated from glycerol stocks (33%, −80°C) and routinely cultured under aerobic conditions at 37°C in a 1% (vol/vol) concentration in sodium lactate broth, which was composed of 10 g liter^−1^ Trypticase soy broth without dextrose (Becton, Dickinson AG, Allschwil, Switzerland); 10 g liter^−1^ yeast extract (Merck, Darmstadt, Germany); 117 mM sodium dl-lactate 60% syrup (Central Drug House, New Delhi, India); 0.25 g liter^−1^ KH_2_PO_4_ (VWR International AG, Dietikon, Switzerland); and 5 mg liter^−1^ MnSO_4_, 4 mg liter^−1^ metronidazole, and 10 mg liter^−1^ kanamycin (all from Sigma-Aldrich, Buchs, Switzerland) in distilled water. Overnight *P. avidum* cultures (200 ml and 1 liter for inoculation of 1 × 10^8^ and 5 × 10^8^ CFU ml^−1^, respectively) were centrifuged at 7,000 rpm for 10 min, the supernatants were discarded, and the bacterial pellets were washed with 0.1 N sodium phosphate buffer (6 g liter^−1^ NaH_2_PO_4_, 7.1 g liter^−1^ Na_2_HPO_4_; both from VWR International AG, Dietikon, Switzerland). The resuspended pellets were centrifuged at 7,000 rpm for 10 min and resuspended in sodium phosphate buffer (10 and 20 ml, respectively) before being added to the test reactors.

*E. limosum* (strain 4119; Laboratory of Food Biotechnology, ETH Zurich), previously isolated from feces of a healthy infant (5), was activated from stabbed agar Hungate stocks (−20°C). The strain was subcultured daily at 3% (vol/vol) in YCFA medium supplemented with 60 mM dl-lactate (Sigma-Aldrich, Buchs, Switzerland) at 37°C under strict anaerobiosis using Hungate tubes flushed with CO_2_ (42, 49). Twenty Hungate tubes containing 10 ml of overnight *E. limosum* cultures were prepared for inoculation of 10^8^ CFU ml^−1^, by centrifugation at 2,000 rpm for 20 min and resuspension in 8 ml of prereduced peptone water (10 g liter^−1^ peptone, 5 g liter^−1^ sodium chloride) before being used to inoculate the reactors. The purity of *P. avidum* and *E. limosum* cultures was checked via Gram staining.

### Fecal inoculum and immobilization.

Two continuous colonic fermentation experiments were performed independently. Fresh fecal samples were obtained from healthy 2-month-old infants born without congenital disease. Because the composition of human milk is very complex and hence difficult to mimic *in vitro*, both infants selected for this study had been fed exclusively with infant formula (see Table S1 in the supplemental material). Exclusion criteria were variables known to affect the balance of the infant gut microbiota, including preterm birth, antibiotic usage, and gastrointestinal and immunological disorders during the neonatal period. The study was exempted by the Ethics Committee of ETH Zurich because the fecal sample collection was noninvasive and not in terms of intervention. Informed written consent was obtained from the mothers on behalf of the infants.

The fecal sample (ca. 5 g) was collected from diapers, immediately suspended in prereduced peptone water (10 g liter^−1^ peptone, 5 g liter^−1^ sodium chloride), transferred into a gastight anaerobic jar containing a CO_2_-generating system (Anaerocult A; VWR International AG, Dietikon, Switzerland), and transported at 4°C for processing, immobilization, and reactor inoculation within 3 h of defecation. Immediately upon receipt, the fecal sample was transferred to an anaerobic chamber and immobilized in 1- to 2-mm-diameter gel beads composed of 2.5% (wt/vol) gellan gum, 0.25% (wt/vol) xanthan gum, and 0.2% (wt/vol) sodium citrate as previously described (17).

### Experiment setup and fermentation procedures.

The fermentation medium was based on the composition designed previously to mimic the chyme entering the colon of 6-month-old infants (17, 50). The medium contained the following (g liter^−1^): lactose (6.4), casein (0.5), whey protein (8.1), peptone (0.5), Bacto tryptone (0.5), mucin (4), yeast extract (2.5), cysteine (0.8), bile salts (0.05), KH_2_PO_4_ (0.5), NaHCO_3_ (1.5), NaCl (4.5), KCl (4.5), MgSO_4_·7H_2_O (1.25), CaCl_2_·2H_2_O (0.1), FeSO_4_·7H_2_O (0.005), hemin (0.01), Tween 80 (1), and vitamin solution. The vitamin solution contained the following (mg liter^−1^): pyridoxine-HCl (100), 4-aminobenzoic acid (PABA) (50), nicotinic acid (50), biotin (4), folic acid (4), cyanocobalamin (5), thiamine (50), riboflavin (50), phylloquinone (0.15), menadione (2), and d-pantothenic acid (100). The nutritive medium was freshly prepared daily, autoclaved, and stored at 4°C under stirring until use. All components were from Sigma-Aldrich (Buchs, Switzerland), except for whey protein (Emmi, Dagmersellen, Switzerland), peptone (Oxoid AG, Pratteln, Switzerland), Bacto tryptone (Becton, Dickinson AG, Allschwil, Switzerland), bile salts (Oxoid AG, Pratteln, Switzerland), and KH_2_PO_4_ (VWR International AG, Dietikon, Switzerland).

The PolyFermS continuous fermentation model used in this study was designed to mimic conditions in the proximal colon of a 2-month-old formula-fed infant. The fermentation setup consisted of a first reactor with a working volume of 200 ml inoculated with 60 ml (30%, vol/vol) fecal beads from the respective donor (IR), which was connected to a control reactor (CR) and four test reactors (TRs) (Fig. 1). All TRs and the CR (200-ml working volume) were continuously inoculated with 5% (vol/vol) fermentation effluent from the IR and fed with 95% fresh medium. To maintain anaerobiosis, all reactor headspaces were constantly flushed with CO_2_. Temperature was set at 37°C, stirring speed was set at 180 rpm, and pH was maintained automatically at 6.0 by adding 2.5 M NaOH.

Initial batch fermentations were carried out at a temperature of 37°C and a pH of 6.0 with stirring (180 rpm) to colonize beads in the IR. During colonization (days 1 and 2), fermentation effluent was replaced by fresh medium every 12 h (17). Afterward, the IR was switched to continuous mode at a flow rate of 40 ml h^−1^, corresponding to a mean RT of 5 h. This flow rate simulated the transit time in the infant proximal colon, which is estimated to be a total transit time of 17.4 h in formula-fed infants aged 113 days (33). After an initial IR stabilization of 5 or 7 days for F1 and F2, respectively, the CR and TRs were connected and operated in continuous mode with the same proximal colon conditions as the IR.

The IR and CR were operated with constant conditions of pH 6.0 and 5-h RT throughout the fermentation time, which was 79 and 57 days for F1 and F2, respectively. Detailed experimental conditions for the two PolyFermS fermentations are depicted in Fig. 1b. After initial stabilization times of 9 and 11 days in F1 and F2, respectively, the fermentations were divided into two periods. During period 1, the effects of pH and RT were studied, while the effects of lactate and LUB on composition and activity of infant gut microbiota were investigated during period 2. Each period consisted of stabilization at pH 6 and a 5-h RT, which was followed by treatment. During treatment 1, combinations of pH (5 or 7) and RT (5 h or 10 h) were assigned to TRs. The pHs (5.0 and 7.0) were chosen to simulate the colonic pH of breast-fed (fecal pH of 5.1 to 5.4 in the first 6 weeks) and formula-fed (fecal pH of 7.0 to 8.2 from the second to the fifth week; fecal pH of 6.4 after the fifth week) infants, respectively (19). During treatment 2, dl-lactate was added in all TRs to achieve a concentration of 60 mM, with or without daily addition of *E. limosum* (10^8^ CFU ml^−1^) and *P. avidum* (1 × 10^8^ or 5 × 10^8^ CFU ml^−1^).

Sampling of effluents from all reactors was performed daily. The sample supernatant (10,000 rpm for 10 min) was used for HPLC analysis, while the pellet was stored at −80°C for DNA extraction. HPLC and qPCR were performed on samples collected during the last 3 days of each stabilization and treatment. MiSeq sequencing was performed on pooled samples collected during the last 2 days of the periods. Plate counts of *P. avidum* were performed in triplicate on samples collected during the last 3 days of stabilization, *P. avidum* treatment, and posttreatment periods (F2).

### Sampling and analysis. (i) DNA extraction.

Total genomic DNA was extracted from 200 mg fresh infant feces and the pellet from 2 ml of fermentation effluent samples using the FastDNA Spin kit for soil (MP Biomedicals, Illkirch, France) according to the manufacturer’s instructions. DNA concentration and quality were assessed by absorbance measurements at 260 nm on a NanoDrop ND-1000 spectrophotometer (Witec AG, Littau, Switzerland), and samples were stored at −20°C before qPCR and MiSeq sequencing analyses.

### (ii) qPCR analysis.

qPCR was performed using an ABI Prism 7500 PCR sequence detection system (Applied Biosystems, Zug, Switzerland). Specific primers targeting predominant bacterial groups or species in the infant gut were used at a final concentration of 0.2 μM (see Table S1 in the supplemental material). Amplification conditions were described previously (4).

### (iii) MiSeq sequencing analysis.

V3-V4 amplicons were prepared using specific forward primer F340 (5′-CCTACGGRAGGCAGCAG-3′) and reverse primer R805 (5′-GGACTACHVGGGTWTCTAAT-3′). Illumina MiSeq sequencing analyses of fecal and effluent samples were carried out at Genotoul (Toulouse, France). Thermocycling was performed with an initial step at 94°C for 60 s, followed by 30 cycles of denaturation at 94°C for 60 s, annealing at 65°C for 60 s, and elongation at 72°C for 60 s, with a final elongation of 10 min at 72°C. The raw data set containing paired-end reads with corresponding quality scores was merged and trimmed using settings as previously mentioned (51). Quantitative Insight Into Microbial Ecology (QIIME) open source software (1.7.0 and 1.8.0) was used for subsequent analysis steps. Purging the data set from chimeric reads and constructing *de novo* operational taxonomic units (OTU) were conducted using the UPARSE pipeline. The HIT 16S rRNA gene collection was used as a reference database.

### Enumeration of *P. avidum*.

Due to the lack of specific primers for *Propionibacterium* amplification by qPCR, *P. avidum* was enumerated in duplicate by plating 100 μl of effluent sample, which had been serially diluted 10-fold, on 1.5% sodium lactate agar supplemented with metronidazole (4 mg liter^−1^) and kanamycin (10 mg liter^−1^) (both from Sigma-Aldrich, Buchs, Switzerland) (52). Antibiotics were used to obtain a higher degree of selectivity for *Propionibacterium* spp., as metronidazole is active against other anaerobic microorganisms (53), such as *Veillonella* species (54), and kanamycin inhibits most Gram-negative (such as Escherichia coli) and some Gram-positive bacteria (55, 56). A combination of kanamycin and metronidazole allows differentiation of *P. avidum*, which forms smooth, cream- to orange-colored convex and circular colonies of various sizes (57). Plates were incubated for 5 days in anaerobic jars at 37°C, and cell counts were reported as log CFU ml^−1^ effluent.

### Metabolite analysis.

The concentrations of SCFAs (acetate, propionate, butyrate, valerate, isobutyrate, and isovalerate), formate, and dl-lactate in effluent samples from all reactors were determined by HPLC analysis. Supernatants from effluent samples were passed through 0.45-μm nylon HPLC filters (Infochroma AG, Zug, Switzerland) before injection. HPLC analysis (Thermo Fisher Scientific Inc. Accela, Wohlen, Switzerland) was performed as described previously (4). Data were expressed as mmol liter^−1^ effluent (mM).

l-Lactate concentration was measured by an enzymatic kit according to the manufacturer’s instructions (Megazyme, Bray, Co. Wicklow, Ireland). d-Lactate concentration was determined by subtracting l-lactate concentration from total dl-lactate concentration.

### Statistical analysis.

Statistical analysis was done using IBM SPSS Statistics 20.0 (IBM Inc., Chicago, IL, USA). qPCR (log_10_-transformed) and HPLC data were expressed as the mean results ± SD for the last 3 days of each fermentation period and compared pairwise between stabilization and treatment within each TR, using repeated-measures ANOVA. Comparisons between reactors within each fermentation period were performed using ANOVA after testing for normal distribution using the Shapiro-Wilk test.

We combined SCFA concentrations and bacterial population levels from the two fermentations for statistical analysis as follows. Differences (delta) between treatment and stabilization period within each reactor were calculated for each combination of 3 measurement days, resulting in 9 delta values per fermentation. Delta values between treatment (TR1 to TR4) and control (CR) reactors were compared using the Wilcoxon rank sum test with false-discovery rate correction. Pairwise comparisons of SCFA concentrations and bacterial population levels between each treatment reactor (TR1 to TR4) and control reactor (CR) during stabilization periods were carried out using the Wilcoxon rank sum test with false-discovery rate correction. For all tests, *P* values < 0.05 were considered significant.

### Data availability.

The sequence data reported in this paper have been deposited in the European Nucleotide Archive database (accession no. PRJEB32244).
