# Supplementary material for: Lactate Metabolism Is Strongly Modulated by Fecal Inoculum, pH, and Retention Time in PolyFermS Continuous Colonic Fermentation Models Mimicking Young Infant Proximal Colon
Source: mSystems. 2019 May 28;4(4):e00264-18. doi: 10.1128/mSystems.00264-18 (PMC6538849; doi:10.1128/mSystems.00264-18)
Supplement: TABLE S1 [file mSystems.00264-18-st001.docx]

**Table S1**

|  |  | **Donor 1** | **Donor 2** |
| --- | --- | --- | --- |
|  | Gender | Female | Male |
|  | Gestational age (weeks) | 38 | 39 |
|  | Mode of delivery | C-section | Vaginal |
|  | Birth weight (gram) | 2780 | 2960 |
|  | Age (days) | 67 | 70 |
|  | Breast-feeding | No | No |
|  | Formula-feeding | Yes | Yes |
|  | Infantile colic | No | No |

| Name | Sequence 5’-3’ | Target gene | Reference |
| --- | --- | --- | --- |
| Eco1457F | CAT TGA CGT TAC CCG CAG AAG AAG C | Enterobacteriaceae | (Bartosch et al., 2004) |
| Eco1652R | CTC TAC GAG ACT CAA GCT TGC |  |  |
| Firm934F | GGA GYA TGT GGT TTA ATT CGA AGC A | Firmicutes | (Guo et al., 2008) |
| Firm1060R | AGC TGA CGA CAA CCA TGC AC |  |  |
| Bac303F | GAA GGT CCC CCA CAT TG | *Bacteroides* spp. | (Ramirez-Farias et al., 2009) |
| Bfr-Femrev | CGC KAC TTG GCT GGT TCAG |  |  |
| xfp-fw | ATCTTCGGACCBGAYGAGAC | *Bifidobacterium* spp. | (Cleusix et al., 2010) |
| xfp-rv | CGATVACGTGVACGAAGGAC |  |  |
| Vspp - F | AYCAACCTGCCCTTCAGA | *Veillonella* spp. | (Price et al., 2007) |
| Vspp - R | CGTCCCGATTAACAGAGCTT |  |  |
| F_Lacto 05 | AGC AGT AGG GAA TCT TCC A | *Lactobacillus*/ *Leuconostoc*/ | (Furet et al., 2009) |
| R_Lacto 04 | CGC CAC TGG TGT TCY TCC ATA TA | *Pediococcus* spp. |  |
| Eli1 | GGC TTG CTG GAC AAA TAC TG | *E. limosum* | (Wang et al., 1996) |
| Eli2 | CTA GGC TCG TCA GAA GGA TG |  |  |

**a**

**b**
